# *APOE* polymorphism and its effect on plasma C-reactive protein levels in a large general population sample

**DOI:** 10.1016/j.humimm.2010.01.008

**Published:** 2010-03

**Authors:** Jaroslav A. Hubacek, Anne Peasey, Hynek Pikhart, Petr Stavek, Ruzena Kubinova, Michael Marmot, Martin Bobak

**Affiliations:** a Institute of Clinical and Experimental Medicine, Prague, Czech Republic; b Centre for Cardiovascular Research, Prague, Czech Republic; c South Bohemia University, Faculty of Public Health and Social Studies, Ceske Budejovice, Czech Republic; d Department of Epidemiology and Public Health, University College London, London, UK; e National Institute of Public Health, Prague, Czech Republic

**Keywords:** *APOE*, C-reactive protein, Polymorphism, Population study, Inflammation

## Abstract

The published data remain inconsistent on association between apolipoprotein E (*APOE*) gene variations and plasma levels of C-reactive protein (CRP), mainly because of low statistical power of previous studies. To clarify this question, we analyzed data from large population sample of randomly selected individuals from seven Czech towns (2,886 males and 3,344 females, the HAPIEE [Health, Alcohol, and Psychosocial factors In Eastern Europe] study). In both males and females, the lowest levels of plasma hsCRP were observed in the carriers of the *APOE ε4ε4* and *ε4ε3* genotypes. The median (interquartile range, IQR) concentration of hsCRP in carriers of the most common *APOE ε3ε3* genotype (two-thirds of participants) was 1.13 mg/l (IQR, 0.56–2.33) in men and 1.23 mg/l (IQR, 0.61–2.65) in women, compared with 0.72 mg/l (IQR, 0.61–0.86) in male and 0.72 mg/l (IQR, 0.61–0.85) in female carriers of *APOE ε4ε3/ε4ε4* genotypes; the differences were statistically significant (*p* < 0.001). The association between *APOE* and CRP was not materially affected by adjustment for age, sex, history of cardiovascular disease, or cardiovascular risk factors. This study, the largest to date, provides robust evidence of an association between plasma hsCRP and the *APOE* genotype, an association not explained by history of cardiovascular disease nor its risk factors.

## 1 Introduction

There is extensive published data on the potential effect of low-grade inflammation in the vascular endothelium on atherosclerosis development [1]. C-reactive protein (CRP), the acute phase serum protein produced by liver, has often been used as an overall marker of inflammation. Elevated plasma levels of CRP have been repeatedly found in patients with cardiovascular disease (CVD) and myocardial infarction, and it has been proposed that CRP is an independent risk factor of CVD [2,3], although the pathogenic and clinical significance of CRP remains controversial [4]. In addition to CVD, plasma levels of CRP have been also found to correlate with CVD risk factors, such as body mass index (BMI), lipid parameters, smoking, and dietary habits [5]. Similarly to other risk factors of atherosclerosis development, plasma levels of CRP are partially genetically determined [6–9].

Apolipoprotein E (*APOE*, gene ID 348, OMIM acc Number 107741) is a multifunctional protein that plays a key role in the metabolism of plasma lipids and is found on the surface of the triglyceride rich lipoproteins. Three common *APOE* alleles (ε*2*, *ε3*, and *ε4*) and many rare mutations have been described; the frequencies of these variants differ between populations [10–13]. In general, individuals carrying the *ε4* allele [Cys112 → Arg, rs429358] have higher total cholesterol levels and persons carrying the *ε2* allele [Arg158 → Cys, rs7412] have lower cholesterol than those carrying the most common *ε3ε3* genotype. The role of the apolipoprotein E, regulated by the *APOE* gene, in the inflammatory response has also been suggested [14], and there is some evidence to support such an effect from studies in both animal models and humans [14,15]. Although data on individual variants are not entirely consistent, a pro-inflammatory state seems associated with *APOE ε4* allele [14]; the same allele is associated with severe sepsis in surgical patients [16].

A possible, but physiologically unexplained, association has been suggested between the common *APOE* polymorphism and plasma level of CRP. However, the results to date are contradictory. Although initial observations suggested that the highest plasma levels of CRP are in *APOE ε3/e3* carriers and the lowest levels in *APOE ε4/e4* and *APOE ε4/e2* carriers [17], subsequent studies were not entirely consistent, as shown in Table 1 [18–35]. A large part of the apparent inconsistencies is due to the very low statistical power of most studies (with a few exceptions), the use of various preselected subgroups (*e.g.*, patients with dyslipidemias or CVD, diabetics, or elderly subjects) and unclear methodology. Clarification of the association between *APOE* genotype and CRP is important for at least two reasons. First, the biologic mechanisms responsible for the association between *APOE* genotype and CVD remain unclear; examining whether associations between *APOE* and potential cardiovascular risk factors (such as CRP) mirror the association of *APOE* with CVD is, therefore, helpful in elucidating the causal pathway. Second, it has long been recognized that apolipoprotein E, and *APOE* genotype, affect a broad range of biologic functions [36], and there is interest in the effects of *APOE* on inflammatory response rate [14].

In this article, we used data from a large population-based study with sufficient statistical power, to investigate differences in plasma CRP levels by *APOE* genotype, and whether such differences can be explained by history of CVD or its risk factors.

## 2 Subjects and methods

### 2.1 Study subjects

We used data from the Czech part of the HAPIEE (Health, Alcohol, and Psychosocial factors In Eastern Europe) study. Details of the study have been described elsewhere [37]. Briefly, random population samples of men and women aged 45–69 years from seven Czech towns (Jihlava, Havirov, Hradec Kralové, Karvina, Kromeriz, Liberec, and Ustí nad Labem) were invited to complete a questionnaire and to attend an examination in a clinic. A total of 8,856 persons completed the questionnaire (response rate: 55%), of whom 7,260 attended the examination, 6,920 provided blood sample, and DNA sample was available on 6,748 individuals.

### 2.2 Measurements

High-sensitivity (hs) CRP was measured immunoturbidimetrically by the WHO Lipid Reference Centre in Prague, using the Roche COBAS (Mannheim, Germany), MIRA autoanalyzer and reagents from Orion Diagnostica, Espoo, Finland (kit no. 68025). DNA was isolated from frozen whole blood using the standard salting-out method [38]. Genotyping of the *APOE* gene was performed as described previously [39]. The frequencies of the individual genotypes are similar to findings in neighboring populations [11], and deviation from the Hardy–Weinberg equilibrium was not statistically significant (*p*-values were 0.13 in men, 0.22 in women, and 0.07 in both genders combined). In seven individuals, a rare *APOE* mutation Arg136Cys was detected [40], and these individuals were excluded from the study. In further 73 individuals, the genotyping failed even when repeated three times. A total of 6,492 persons had valid data, both on CRP level and *APOE* genotype.

### 2.3 Statistical analyses

We further excluded subjects who had hsCRP ≥ 10 mg/l CRP levels (*n* = 262) from the analysis because of a possible acute infection. The present analyses are thus based on 6,230 subjects (2,886 males and 3,344 females) with full data on CRP and *APOE* and with hsCRP concentrations <10 mg/l.

Because the distribution of hsCRP levels is highly skewed, to the extent that even logarithmically transformed values are highly asymmetrically distributed, we used nonparametric methods. First, we show, for each *APOE* genotype, and within each sex, the median and interquartile range (IQR) of hsCRP concentrations. Second, crude differences in hsCRP levels by *APOE* genotype were assessed by the Kruskal–Wallis nonparametric test, separately for men and women. Finally, because the differences in hsCRP by *APOE* genotypes were similar in men and women, data from both genders were pooled, and the relationship between *APOE* genotype and quartile of hsCRP was estimated using ordered logistic regression. This approach, while not making assumption about the distribution of CRP, allows multivariate analysis and therefore adjustment for covariates; we controlled for age, sex, history of myocardial infarction, angina and stroke (each coded as 0/1), current smoking, total cholesterol, BMI (kg/m^2^), and hypertension (systolic/diastolic blood pressure ≥140/85 mm Hg or antihypertensive medication in the last 2 weeks). The odds ratio derived from ordered logistic regression can be interpreted as the weighted mean of (i) odds of CRP quartile 1 vs. quartiles 2–4, (ii) odds of quartiles 1–2 vs. quartiles 3–4, and (iii) odds of quartiles 1–3 vs. quartile 4, associated with a given genotype. The proportional odds assumption was fulfilled (the *p*-value from the approximate likelihood-ratio test of proportionality of odds across CRP quartile response categories was 0.133).

## 3 Results

Descriptive characteristics of the subjects included in the analysis are summarized in Table 2. The median CRP levels were slightly higher in men than in women (*p* < 0.001) and, as expected, CRP was positively correlated with age, BMI, and smoking (not shown in table). The distribution of the *APOE* genotypes in individuals excluded because of CRP concentration > =10 mg/l did not differ from the general population (*APOE ε2ε2* 0.8%, *APOE ε2ε3* 13.7%, *APOE ε3ε3* 65.3%, *APOE ε4ε3* 16.8%, *APOE ε4ε2* 2.7%, and *APOE ε4ε4* 0.8%, not shown in table).

The plasma levels hsCRP by the *APOE* genotypes are summarized in Table 3. Serum levels of hsCRP differed highly significantly between *APOE* genotypes, both by all six individual genotypes and by four groups defined by the presence of the *APOE* alleles, that is, carriers of the *APOE ε2* allele, *APOE ε3ε3* homozygotes, and *APOE ε4* carriers. The *APOE ε2ε4* genotype is also reported separately because of the uncertainty about pooling with other genotypes. The differences were of similar magnitude in males and females. In males, *APOE ε3ε3* homozygotes had the highest median serum hsCRP; similarly, females with *APOE ε3ε3* genotype also had high CRP (only very slightly lower median than *APOE ε4ε2* heterozygotes); even in such a large study, however, the numbers of individuals with specific uncommon genotypes within each sex were insufficient to provide statistically reliable estimates of hsCRP levels.

Given the similarity of associations in men and women (and the interactions between sex and *APOE* were not statistically significant), we pooled the data from both genders, and we used ordered logistic regression to assess the association between *APOE* genotype and hsCRP after controlling for covariates (Table 4). The associations remained strong and highly statistically significant after adjustment for the age, history of CVD, smoking, BMI, total cholesterol, and hypertension. In addition to lower hsCRP in the combined *APOE ε4* group, both *APOE ε4ε3* and *APOE ε4ε4* genotypes had statistically lower levels of hsCRP than *APOE ε3ε3* homozygotes in these pooled analyses (because of increased statistical power, compared with sex-specific analyses).

## 4 Discussion

The present study, the largest on this subject to date, found that the serum hsCRP is independently determined by the common genetic polymorphisms within the *APOE* gene. Given the large size (and hence, high statistical power) of this study, these findings provide robust evidence that the *APOE ε4* allele is associated with low levels of plasma hsCRP.

Previous studies on this subject did not yield results that were not entirely consistent. In some populations, both *ε2* and *ε3* carriers were reported to have the highest CRP [17,18]. Some studies of elderly subjects showed either the highest levels of CRP in *APOE ε2* carriers [19,21] or no effect of *APOE* genotype on plasma CRP at all [22]. A study in younger adult females found the highest hsCRP levels in carriers of the *APOE ε4* allele [23]. In a recent genome-wide association study, CRP levels had a significant genetic component and *APOE* was among genes detected to have a significant effect on plasma CRP; however, CRP values by the specific alleles were not available [8].

The discrepancies in previous findings are mainly due to the low statistical power of most previous studies. In addition, many studies analyzed subjects who included patient's relatives or preselected subgroups, such as dyslipidemic subjects [17,21,27], ethnically nonhomogenous populations [20], the elderly [19,22], or families of patients [24,26]. There were also methodological inconsistencies; for example, many papers do not specify whether extreme hsCRP levels were excluded from the analyses (because of the concerns about the presence of acute infection) or the grouping of *APOE* genotypes differs between papers. Inappropriate statistics may have also contributed to the heterogeneous findings—most studies reported geometrical means of hsCRP but, given the extremely skewed distribution of hsCRP, the use of parametric methods is likely to produce misleading results. Overall, previous data mostly suggest low CRP levels in carriers of ε4 compared with ε3ε3, but the inconsistencies in reporting allow neither reliable conclusions nor formal meta-analysis without obtaining the raw data.

Our study overcame many of these limitations. First, its size (roughly equivalent to half of all previous published studies combined) provided sufficient statistical power to study not only groups of genotypes, but specific genotypes separately. Second, contrary to many previous investigations, our study recruited a random general (and ethnically homogenous) population sample, rather than patients selected for a particular condition, such as candidates for statin treatment, diabetics, or different ethnicities. Third, our study sample was slightly younger than many previous studies (the mean age in many studies was considerably higher than sixty years); as age is an important predictor of CRP, studies in younger subjects are particularly useful. Fourth, we excluded subjects with high CRP levels, and thus limited misclassification because of possible acute infection, and used nonparametric methods that do not make assumptions about distribution of CRP. Finally, our subjects were phenotypically well characterized, enabling us to discount history of CVD and risk factors for CVD as possible explanations for the relation between *APOE* genotype and hsCRP.

The biologic basis for the relationship between *APOE* and hsCRP is not clear. Given that both plasma apolipoprotein E and CRP come from the same source in organism, the liver, the expression of these proteins could be regulated by similar factors, but no experimental evidence has been published so far.

The present results, consistent with most previously published data, highlight an important contradiction. On the one hand, individuals with elevated levels of the hsCRP are at increased risk of mortality and morbidity from multiple disease states, including myocardial infarction, stroke, diabetes, metabolic syndrome [3], and end stage renal disease [14]. By contrast, *APOE ε4* is associated with increased risk of CVD and end stage renal disease [41]. Yet, the CRP levels are lower in carriers of *APOE* ε4, which is the “risky” genotype for CVD, and high concentrations of hsCRP, proposed to be a risk factor of CVD, are not associated with the *APOE ε4* allele as would be expected. To complicate the situation further, there is an inverse association between plasma apolipoprotein E concentration and CRP levels [42], and plasma apolipoprotein concentrations seem to be higher in *APOE ε2* carriers and lower in *APOE ε4* carriers [43]. This suggests that the effects of *APOE* and CRP operate by different and mutually independent mechanisms, or that the *APOE* genotype is associated with, but is not a causal determinant of plasma CRP levels.

It is possible that slightly elevated CRP levels, based on genetic predisposition, rather than inflammatory status, account for the elevated risk of vascular disease. Indeed, a large prospective study has shown that variants in the CRP gene are associated with marked increase of CRP levels, but these polymorphisms were not associated with increased risk of vascular disease [44]. This would allow for the possibility that adverse effects of increases in CRP levels are limited to inflammation induced environmentally, rather than genetically.

Our study was not designed to investigate the important question of the etiological role of CRP in CVD. However, we have clearly demonstrated that the *APOE* genotype influences CRP concentrations. Future studies are needed to clarify the implication of this association for the risk of common diseases, such as vascular diseases.

## Figures and Tables

**Table 1:** Previous population-based studies on *APOE* genotype and CRP levels

Main author	Year	Study population	Measure of CRP levels	Number of persons	Main findings	Differences in CRP statistically significant?
Kravitz et al. [33]	2009	Persons aged 90+	3 groups	227	No association	No
Angelopoulos et al. [32]	2008	Healthy volunteers	Geometric mean	117	Lower CRP in ε3/ ε4	Marginally
Haan et al. [31]	2008	Elderly Latinos	Median	1445	Lower CRP in ε3/ ε4	Yes
Gronroos et al. [23]	2008	General young population	Geometric mean	1221	Lower in ε4 in men, not in females	Yes/No
Berrahmoune et al. [24]	2007	General population	Geometric mean	1223	Lower CRP in ε4	Yes
Park et al. [34]	2007	Healthy controls	Arithmetic mean	119	No association	No
Kahri et al. [26]	2006	Low HDL & normo-lipidaemic subjects	Geometric mean	368	CRP lower in ε4	Yes
Tziakas et al. [29]	2006	Patients with angina & acute coronary syndrome	Non-parametric	166	CRP lower in ε4	Yes
Ravaglia et al. [30]	2006	General 65+	Binary	671	CRP lower in ε4	No
Lange et al. [20]	2006	Diabetics	Geometric mean	241	CRP lower in ε4	Marginally
Mooijaart et al. [22]	2006	Persons aged 85+	Geometric mean	594	CRP lower in ε4	Yes
Chasman et al. [25]	2006	General population	Geometric mean	2053	CRP lower in ε4	Yes
Rontu et al. [19]	2006	90+	Geometric mean	291	CRP lower in ε4	Yes
Eiriksdotrir et al. [21]	2006	Older general population	Geometric mean	2251	CRP lower in ε4	Yes
Paschos et al. [28]	2005	Dyslipidaemic	Median	50	CRP lower in ε4	No
Judson et al. [27]	2004	Dislipidaemic	Geometric mean	600	CRP lower in ε4	yes
Marz et al. [18]	2004	Patients with CHD and healthy controls	Geometric mean	1309	CRP lower in ε4	Yes
Austin et al. [35]	2004	Japanese Americans from 68 kindreds	Geometric mean	558	CRP similar in ε4 and ε3/ ε3 but lower than in ε2	Marginally
Manttari et al. [17]	2001	Cases of MI and controls	Arithmetic mean	177	CRP lower in ε4	Yes

Note: Studies were identified in PubMed using the following combinations of search terms: (“apoe” or “apo e” or “apoliporotein e”) and (“genotype” or “gene” or “polymorphisms”) and (“crp” or “c-reactive protein”).

**Table 2:** Basic characteristics of subjects included in the analysis

	Men	Women
N	2886	3344
Age, mean (SD), yr	58.2 (7.2)	57.4 (7.2)
Total cholesterol, mean (SD), mmol/l	5.62 (1.04)	5.82 (1.04)
BMI, mean (SD), kg/m^2^	28.2 (3.9)	27.9 (4.9)
Hypertension, %	72.5%	57.9%
Current smoking prevalence, %	25.9%	20.3%
History of myocardial infarction, %	7.8%	2.4%
History of angina, %	5.1%	3.3%
History of stroke, %	3.5%	2.5%
CRP, median (IQR), mg/l	1.08 (0.56, 2.19)	1.19 (0.57, 2.61)

**Table 3:** Median (IQR) concentrations of CRP by *APOE* genotype and odds ratios (95% confidence intervals) from ordered logistic regression (with quartiles of CRP as dependent variable), by sex

*APOE*	N	%	Median (IQR) of CRP concentrations (mg/l)	Odds ratio (95% CI)
Men				
All	2886	100	1.08 (0.56, 2.19)	—
22	15	0.5	0.82 (0.45, 2.83)	0.76 (0.29–1.97)
32	309	10.7	1.12 (0.57, 2.16)	0.97 (0.78–1.20)
33	1926	66.7	1.13 (0.56, 2.33)	1.0 (reference)
42	56	1.9	1.10 (0.60, 2.01)	0.93 (0.58–1.47)
43	551	19.1	0.91 (0.52, 1.91)	0.77 (0.65–0.92)
44	29	1.0	0.61 (0.35, 1.19)	0.34 (0.17–0.68)
			*p =* 0.0004^a^	
33	1926	66.7	1.13 (0.56, 2.33)	1.0 (reference)
22 + 32	324	11.2	1.12 (0.56, 2.17)	0.96 (0.78–1.18)
43 + 44	580	20.1	0.90 (0.49, 1.88)	0.75 (0.63–0.88)
42	56	1.9	1.10 (0.60, 2.01)	0.93 (0.58–1.47)
			*p =* 0.0004^a^	
Women				
All	3344	100	1.19 (0.57, 2.61)	—
22	27	0.8	0.81 (0.52, 2.33)	0.66 (0.33–1.33)
32	399	11.9	1.07 (0.62, 2.42)	0.88 (0.73–1.06)
33	2200	65.8	1.25 (0.61, 2.65)	1.0 (reference)
42	66	2.0	1.33 (0.58, 3.46)	1.15 (0.74–1.79)
43	604	18.1	0.98 (0.48, 2.38)	0.70 (0.60–0.81)
44	48	1.4	0.91 (0.49, 2.64)	0.62 (0.36–1.08)
			*p =* 0.0052^a^	
33	2200	65.8	1.25 (0.61, 2.65)	1.0 (reference)
22 + 32	426	12.7	1.07 (0.61, 2.40)	0.86 (0.72–1.03)
43 + 44	652	19.5	0.97 (0.48, 2.40)	0.70 (0.60–0.83)
42	66	2.0	1.33 (0.58, 3.46)	1.15 (0.74–1.79)
			*p =* 0.0010^a^	

a Kruskal-Wallis non-parametric test.

**Table 4:** Crude and adjusted odds ratios (95% confidence intervals) by *APOE* genotype from ordered logistic regression, with quartiles of CRP concentrations as dependent variable; pooled data from men and women

*APOE* genotype	N	%	Crude OR (95% CI)	Adjusted OR (95% CI)^a^
All subjects	6230	100		—
22	42	0.7	0.71 (0.40–1.24)	0.58 (0.31–1.08)
32	708	11.4	0.92 (0.80–1.06)	0.92 (0.79–1.07)
33	4126	66.2	1.0 (reference)	1.0 (reference)
42	122	1.9	1.03 (0.75–1.42)	1.05 (0.75–1.48)
43	1155	18.5	0.73 (0.65–0.83)	0.69 (0.61–0.78)
44	77	1.2	0.50 (0.33–0.76)	0.47 (0.30–0.73)
33	4126	66.2	1.0	1.0 (reference)
22 + 32	750	12.1	0.91 (0.79–1.04)	0.90 (0.78–1.04)
43 + 44	1232	19.8	0.72 (0.64–0.81)	0.67 (0.60–0.76)
42	122	1.9	1.03 (0.75–1.42)	1.05 (0.75–1.48)

a adjusted for age, sex, total cholesterol, hypertension, body mass index, smoking, and history of angina, myocardial infarction and stroke.
